# Connecting human behaviour, meaning and nature

**DOI:** 10.1098/rstb.2022.0314

**Published:** 2024-04-22

**Authors:** J. M. Anderies, C. Folke

**Affiliations:** ^1^ School of Human Evolution and Social Change and School of Sustainability, Arizona State University, Tempe, AZ 85287, USA; ^2^ Beijer Institute of Ecological Economics and the Anthropocene Laboratory, Royal Swedish Academy of Sciences and Stockholm Resilience Centre, Stockholm University, SE-104 05 Stockholm, Sweden

**Keywords:** biosphere, human behaviour, imagined order, revitalize

## Abstract

Much of the discourse around climate change and the situation of diverse human societies and cultures in the Anthropocene focuses on responding to scientific understanding of the dynamics of the biosphere by adjusting existing institutional and organizational structures. Our emerging scientific understanding of human behaviour and the mechanisms that enable groups to achieve large-scale coordination and cooperation suggests that incrementally adjusting existing institutions and organizations will not be sufficient to confront current global-scale challenges. Specifically, the transaction costs of operating institutions to induce selfish rational actors to consider social welfare in their decision-making are too high. Rather, we highlight the importance of networks of shared stories that become real—imagined orders—that create context, meaning and shared purpose for framing decisions and guiding action. We explore imagined orders that have contributed to bringing global societies to where they are and propose elements of a science-informed imagined order essential to enabling societies to flourish in the Anthropocene biosphere.

This article is part of the theme issue ‘Bringing nature into decision-making’.

## 1. Introduction

As climate change impacts become more apparent, so too has the need for changing the way humans live in the biosphere [1,2]. From a traditional environmental policy perspective, changing the way diverse human communities and societies live in the biosphere means designing institutions and organizations to provide information and use it to regulate or incentivize a certain set of choices by human actors to steer the system toward a socially desirable goal [3]. That is, given our understanding of the workings of biosphere systems, how should institutions be designed, i.e. the rules of the game and organizations to implement those rules, to generate a particular set of social outcomes? This is an extremely challenging task given the complexity of our situation, with globally connected social and economic systems deeply intertwined with the biosphere [4].

The effectiveness of interventions based on this approach depends on how good our model of human behaviour is and our ability to predict how it creates and interacts with higher-level structures across multiple organizational scales [5]. In environmental policy and in behavioural economics in particular, human ‘behaviour' is characterized in terms of the decisions people make in particular contexts. Research has focused on how changes in social, informational and environmental dimensions of decision situations affect peoples' choices. Improvement of our understanding of the links between contexts and decisions is seen as critical for improving our capacity to govern ourselves and our interactions with the environment.

However, how the behaviour of individuals changes with varying contexts is only part of the story. There is more to individual behaviour than a collection of decision-making algorithms deployed in various situations to achieve a narrowly defined goal, e.g. rational, selfish behaviour. Perhaps on short time-scales and in particular contexts such as in the case of the traditional policy problem of managing a clearly bounded resource system with a clearly bounded user community, a model of humans as selfish rational actors is sufficient. However, on large time-scales and in open-ended, uncertain systems such as the organization of the global economy, global social orders and the biosphere, such models are insufficient. In such cases, the decision-making context is ambiguous and difficult to define. Yet humans must and do make decisions in such settings.

When faced with such ambiguity, it is natural to assume that humans may turn to a higher-level, context-independent decision-making framework, e.g. something akin to moral reasoning. But Haidt [6] asks what underlies moral reasoning and argues that work in cognitive science and moral psychology reveals that something yet deeper is at play: moral emotions and intuition. He further argues that moral reasoning is not a rational decision-making process but, rather, a process by which humans justify underlying intuitions [7]. Policy processes concerned with long-run trajectories of human societies in the biosphere must account for overarching ethical and moral systems driven by emotions and intuition that guide human choices. In more technical terms, if social organizational forms, e.g. communities, economic systems and social-ecological systems, are complex adaptive systems driven at their core by emotions and intuition, what are the long-run attractors and how are they reached? And, of course, this begs the question of what factors underlie emotions and intuition.

It has long been argued that social organization understood as selfish rational agents interacting through market exchange and formal institutions cannot account for the larger arc of societal development patterns. Fukuyama [8] suggests that large-scale coordination requires ‘spontaneous sociability' to overcome the transaction costs associated with exchange in formal market systems. Spontaneous sociability relies on trust that emerges endogenously from the intersection of underlying moral codes, institutional arrangements, technology and shared values. Weber [9] famously suggested that religious principles in the form of the Protestant work ethic were critical for providing a basis for spontaneous sociability for organizing economic production and exchange. That is, the shared story of Protestantism provided a basis for people to maintain group identity at larger scales. Fukuyama points out that the triumph of the story of liberal humanist democracies suggests that it is the most robust template for organizational forms [10]. But societies based on this story can develop in many different directions. How are these directions determined?

These ideas suggest that shared stories function on at least two levels: (i) they help grease the wheels of coordination and exchange and (ii) they provide sense-making capacity to cope with ambiguous contexts, e.g. providing heuristics to help processing information/experience through decision-making or emotional responses, and a ‘why' for living. There is a large, interesting and diverse scientific literature on cultural evolution that addresses the many nuances of how stories, as part of culture, interact with our psychology and the reproductive success of individuals and groups within these two broad categories [11–16]. Surveying this literature is beyond the scope of this article. What is important for our discussion is that this work provides a basis for thinking about the evolution of stories and cultural group selection. Stories have a real impact on the success of individuals and groups.

In sociology, anthropology and philosophy, the importance of stories has long been recognized via the notion of social imaginaries or imagined social totalities. These imaginaries are networks of shared stories that underlie inter-subjective realities, neither objective nor subjective, but ‘real' by virtue of the fact that members of a group agree on them. They impact behaviour in very concrete ways whether in ancient indigenous lifeways or the most recent and novel forms of lifestyles and cultures. Imaginaries operate in such a disparate range of activities both at the individual and social level that it is challenging to think of situations in which they don't play some role. Again, a careful review of this rich and extensive literature is beyond the scope of this article, but we can highlight key examples relevant to our discussion here. First, social imaginaries in so far as they incorporate a sense of our normal expectations of one another [17] play a key role in supporting trust which, in turn, is critical for exchange and the functioning of markets. As Arrow [18, p. 357] put it ‘Virtually every commercial transaction has within itself an element of trust…*'.* Second, social imaginaries play a key role in how we value objects [19] vis à vis our status in our imagined communities which, in turn, drives consumption choices. Third, imaginaries play a key role in political processes as tools for coalition building. Political and other powerful actors use historical myths [20], invented traditions [21], or stories related to the role that imagined social totalities should play in their lives to build coalitions for their projects (e.g. to get elected). Because powerful actors have disproportionate capacity to tell stories, their actions can generate narrative wars that act as barriers to meaningful discourse and collective action on pressing societal problems. Fourth, powerful actors may use imaginaries to shape and promote their visions of the future, drive investment in large-scale infrastructure to support those visions, and generate negative unintended consequences for society [22]. Finally, imaginaries help enhance cooperative behaviour, e.g. stories that prime group achievements may increase the willingness of group members to engage in costly cooperation for the benefit of their group [23]. These particular roles of shared narratives are critical for human societies to thrive within the biosphere as they drive our consumption patterns and work both for and against our capacity to engage in costly collective action to address shared problems.

**Figure 1.**
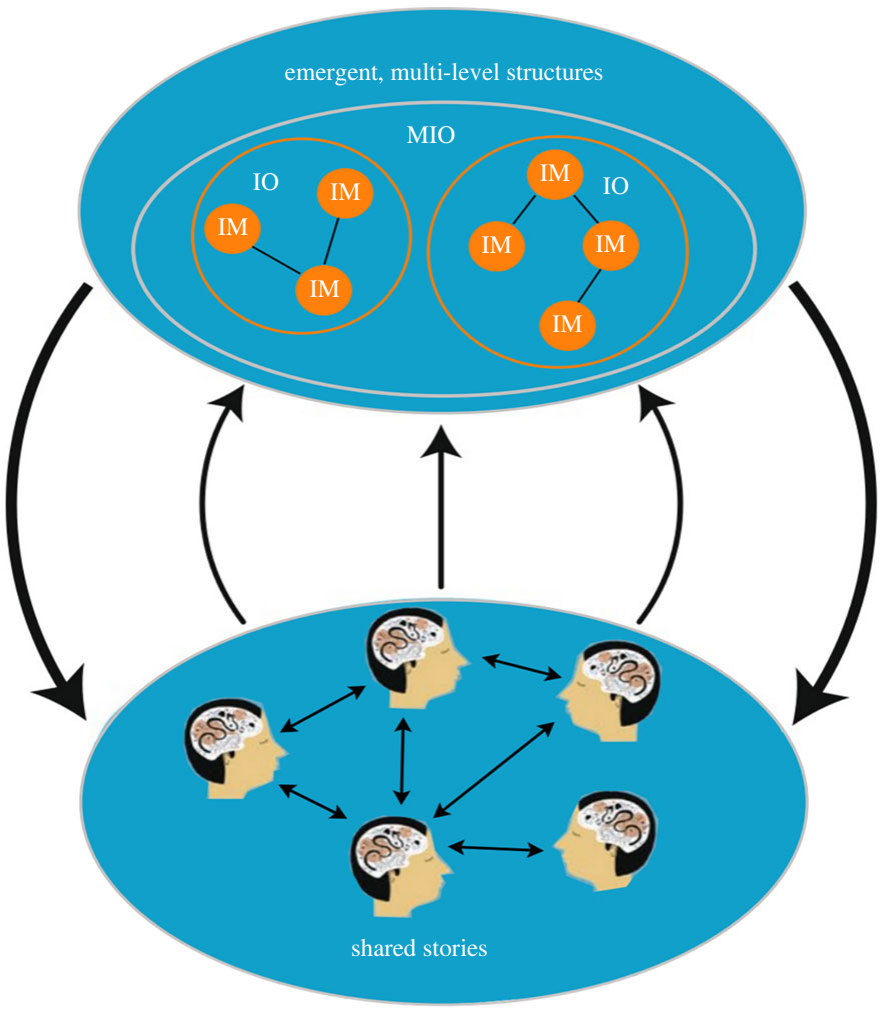
Imagined orders (IO) in the form of networks of nested imaginaries (networks of shared stories that give rise to intersubjective realities (IM) that emerge as multi-level structures that create social orders. Multiple IO can coexist within a higher-level meta-imagined order (MIO). Adapted from Schill *et al*. [5].

In his popular book *Sapiens: A brief history of humankind*, Harari [24] uses the term *imagined order* to refer to a network of imaginaries (shared stories that underlie inter-subjective realities). We adopt this term here as it emphasizes the role of imaginaries in creating and maintaining social orders. Social orders are multi-level structures that emerge from the interactions of individual agents that then impact individual behaviour in an open-ended coevolutionary process of actions (agency) and the creation of context for those actions (structure), as shown in figure 1. In our view, an imagined order represents a meta story in which many other imaginaries can flourish. A meta-story contains basic overall principles, shared purpose and direction, like a meta-attractor, towards which shared stories and networks of stories (imaginaries) self-organize. Complex, culturally and regionally specific stories may flourish and coexist within the meta story, within the imagined order. For example, one important class of imagined order involves a force (or forces) outside of human experience and agency that provides guiding principles for social order and collective action, i.e. a cosmic order. More detailed stories nested within a cosmic order may take many different forms but typically have a clear description of a shared purpose that determines overall social organization and action. Such cosmic orders are instantiated as rituals and taboos in small-scale societies or in major religions that can shape the organization of very large populations. By contrast, the imagined order of liberal humanism focusing on individual liberty and realizing each person's full human potential provides less guidance on a shared purpose. Further, how can a diverse set of imagined orders, each with differing notions of shared purpose, coexist and coordinate action at the level of the biosphere? Perhaps what is needed is a meta-imagined order with very few core principles around universal principles of care and sacrifice towards a shared purpose of living within the biosphere?

Addressing these questions is critical for modern societies. As the global network of societies reaches a scale at which their impacts on the biosphere begin to challenge their very existence, the need to find imagined orders and their associated inter-subjective realities that are capable of addressing the challenges societies face becomes ever more pressing. We first explore the notion of imagined orders as part of the software operating system that runs the interacting hardware (human technologies) and greenware (biosphere) systems. We then explore some characteristics of imagined orders that underlie present forms of social organizations and then use them to turn to the question of creating imagined orders capable of supporting well-being for an envisioned human population of at least nine billion, without destabilizing the biosphere and driving it to a new attractor inhospitable to the human enterprise [25].

## 2. Deepening the view of social-ecological systems: hardware, software and greenware

Models of social orders, i.e. economies and the legal structures that support them, are often based on the assumption of self-interested rational actors operating in specific institutional contexts divorced from the social, cultural and natural environment. We know from recent evidence from behavioural sciences that human behaviour is far more complex and varied than that ascribed to *Homo economicus*. Humans care about fairness [26,27], take others into account in their decisions [28–30] and are intrinsically pro-social [31]. Humans readily engage in punishment of others who have violated social norms, even when such punishment is costly to inflict [13,32]. These features of human behaviour, i.e. prosociality, social sanctioning and control, and our ability to maintain a flexible inventory of symbols with shared meaning have been identified as critical interacting factors in cultural evolution (see [33]).

The question facing designers of environmental policy in a world with different types of behaviour and where institutions themselves mould behaviour is thus much more complex than the problem of getting the institutions right, given rational agents with infinite computing power and perfect foresight. The information processing load is just too high, and humans simply aren't wired to process information in this way. Even accounting for the complexity of human behaviour, getting the institutions right is challenging because they coevolve with preferences and may change social norms or mental models that people use to guide their behaviour (the arrows go both ways in figure 1). At the same time, behaviour coevolves with technology [34,35]. All these dynamically interacting systems are embedded in the biosphere.

The perspective of social-ecological systems was developed to emphasize that humans, irrespective of beliefs, values or culture, are not separated from the ecological systems that support them [36]. And thus, ‘governance' or ‘policy' may be understood as an emergent feature of a complex adaptive system that defines the social-ecological system. From this perspective, the interplay between people and ecosystems is more than just linked, overlapping or interdependent. In this interplay, people and ecosystems are inseparable, as has become ever more evident in the Anthropocene era, where human societies and cultures and our actions now shape the dynamics of the Earth System and its fragile Biosphere at the planetary level. Acknowledging the inseparability of social-ecological systems presents a very different entry point for exploring the challenges of sustainability, bringing to the fore theories of social-ecological emergence, coevolution and diversity [37].

Furthermore, social-ecological systems are part designed and part self-organizing. The designed aspects of social-ecological systems include both ‘hardware' (paths, roads, canals, boats, tools, buildings) and ‘software’ (norms, rules, networks of shared meanings, ‘culture') that entangle humans with the biosphere (greenware). In fact, we suggest that one of the most important aspects of any society is the network of shared stories that can be seen as shared social infrastructure and that serves to solidify large-scale social organizations and to unify natural, hard human-made, soft human-made, human and natural infrastructures in a *coupled infrastructure system*. The structure of this coupled infrastructure system and the stories that get told within it are of course influenced by power structures [22]. But it has also been recognized that transforming towards sustainable futures will require broadening cultural membership by promoting networks of stories that allow for a plurality of values and social inclusion [38,39]. Examples of such broadening are the active engagement with indigenous ontologies and non-Western models of environmentalism [40,41] in several international policy processes [39,42]. In this context, does there exist an imagined order, in the face of diverse and pluralistic belief systems, power structures, values and cultures, which can guide the development of large-scale human organizational forms that are capable of navigating human actions within the capacity of the biosphere and the Earth system to sustain development [4]? Such a meta-imagined order could be interpreted as a core attractor for framing the emergence and transformations toward sustainable futures, as a sort of high-level software that enables mass-scale coordination, collaboration and collective action.

## 3. Stories that got us here

Creating a new imagined order to guide diverse human development pathways in the Anthropocene will require the development of coherent sets of shared ideas about the future [43], drawing their authority from empirical foundations, significant experiences of communities and non-rational roots [44]. Given that the liberal humanist imagined order has been extraordinarily effective in enabling human societies to coordinate at large scales to create material abundance, health, innovation and opportunities for creativity, it is tempting to use it as a basis for imagining new futures. However, liberal humanist societies are encountering problems that they cannot solve and more may be required than changes to this story at the margins. What may be needed is an imagined order that embeds human societies in the biosphere. It is not clear yet exactly what this story is. What dynamic process of story formation and infrastructure investments (the building of economies and societies) lead to sustained wellbeing of people as part of the biosphere? Before turning to this question, we set the stage by describing some elements of imagined orders from the past to gain insight into a story for the future.

The sequence of imagined orders does not imply an historical progression of social organization. There are many paths to complexity in social orders and no clear direction towards increasing complexity or hierarchical forms [45]. Further, we aren't suggesting the orders apply to actual historical cases. We are presenting some ingredients of the imagined orders about how present social structures may have emerged (especially Western notions of social, political and economic organization), stories that still appear in the literature, often implicitly, as the baseline structure that needs to be ‘fixed'. The following imagined orders suggest that more than a fix is required and help us see what the minimum criteria of a new imagined order might be: embeddedness in the biosphere.

### (a) Story 1: Externally imposed cosmic order, no/slow economic growth

In this imagined order, an external deity/force maintains order by providing a moral code and meaning. Earthly society is a reflection of a cosmic plan—whether the battle of good and evil deities or the result of a single benevolent deity. There is no belief in human agency to change the future, and society 100 years from now will look the same as it does today (little or no technological change). The effect of such imagined orders on behaviour is that individuals accept their position, play their role and hope to be rewarded as part of the cosmic order. This leads to stable social forms, with producers and two types of elites: providers of sovereignty and controllers of esoteric knowledge [45–47]. The obvious long-run problem is that population growth and associated consumption patterns eventually strain resources [48]. Of course, migration of some form provides a relief valve, but the system becomes vulnerable to small variations in resource flows and social tensions. This order offers little guidance for the modern context other than attempting to match resource availability to population size.

### (b) Story 2: Growth under liberal humanism

In this imagined order, there is no externally imposed cosmic order. Humanism supports beliefs in the capacity of humans to determine their destiny, solve problems and create wellbeing. Liberalism enforces the right to realize human potential as long as this does not impinge on others' rights to do the same. Without an externally enforced moral code and a definition of meaning, evolutionary aspects of behaviour emerge, e.g. competition for status through material consumption and power. The state is rudderless and meritocratic competition processes can spin out of control, leading to increasing inequality and potential conflict [49]. In the modern context, this imagined order highlights fundamental flaws in modern economic systems that must be addressed but de-emphasizes the potential impacts of resource scarcity.

### (c) Story 3: Growth and collapse under liberal humanism with a resource constraint

Under liberal humanism in a capitalist institutional setting, consumption grows without bound as humans all work to achieve their full potential. Resource constraints are acknowledged but tensions emerge around differing beliefs about whether humans are fundamentally limited in their capacity to overcome them through infrastructure transitions. The central modern example is energy system transitions. A transition to clean energy can be seen as a switch from ‘mining' petroleum with its problems to mining minerals with their problems. Short-run solutions just generate more problems, which eventually pile up beyond our control [50].

### (d) Story 4: Growth under liberal humanism in a race with complexity

In this imagined order, resources are not a limitation. Rather in this case, the culprit is nonlinear dynamics in the natural world and the systems societies create that lead to rapid unpredictable change. Thus, societies are limited by the capacity of the organizations they create to manage ever-increasing social and technological complexity [51,52]. In the modern context, the most prominent example is increasing reliance on artificial intelligence at a faster rate than societies can develop capacity to govern its responsible use [53,54].

### (e) Story 5: Growth under liberal humanism that acknowledges complexity and the biosphere

This is an extension of Story 4 that recognizes that societies are co-creating a complex system with the biosphere. Society is challenged with increasing costs associated with maintaining social, economic and technological complexity, but whether society succumbs to the complexity it creates depends on the biosphere—a complex system with nonlinear dynamics that can shift too fast for humans to respond [25,55,56]. This imagined order is just emerging.

As overly simple as these characterizations are, many features resonate with present-day experiences and historical narratives. Story 1 features in many historical narratives in which dogma dominates and is leveraged by elites. Examples of such social orders (as defined by the imagined order and other infrastructures—both natural and human-made) may have lasted for centuries, but then, the planet was not full of humans and dominated by human actions, and there were always relief valves. Some might argue that citizens of modern industrialized economies are living in Story 4, and, if taken seriously, complex systems science suggests that global society could be at a tipping point. Modern discourse around building international organizations to manage complex interactions with the biosphere suggests that Story 4 still drives thinking in a vicious cycle of creating complexity to deal with complexity.

The alternative as suggested by Harari [53] is that Story 4 goes in a different direction—inequality becomes extreme, with the hyper-wealthy becoming immortal and others becoming irrelevant. But can this future be achieved before Earth System dynamics change so rapidly that human societies can't respond? Can modern globalized societies transition from Story 4 to Story 5? Can societies collaboratively build a coupled infrastructure system, including a robust imagined order, which does not further erode the resilience of the biosphere, our essential piece of infrastructure with which we are intertwined and within which we are embedded? It is to this question that we now turn.

## 4. Stories to get us there: revitalizing the biosphere

Current stories of the Anthropocene situation, amplified by news and media, give a flavour of sustained crisis and long-term sacrifice. Sacrifice is defined by the social order and the story. In liberal humanism, sacrifice means giving up some freedoms and flows related to realizing individuals' concerns, like selfish consumption. There is need for a new imagined order of the human condition in the Anthropocene, an imagined order that shifts from dystopia and sacrifice towards a sustainable future on a livable planet, a story that creates hope and meaning to help support a new imagined order, perhaps a meta-imagined order, to guide the development of society in the Anthropocene. Such a story will have to redefine our economic, social and cultural relationship with the living planet, to make us enearthed [5] and translate this into operational governance and management to enable sustainable futures [57]. In this sense, environmental issues have become issues of how to increase the likelihood for societal development pathways that can generate, sustain and improve human wellbeing and prosperity as part of and in concert with the resilience of the biosphere [4,58].

The challenge is to become stewards of our own future by revitalizing the biosphere. Revitalizing the biosphere includes both how groups govern, collaborate and perform collective action, as well as how such governance, collaboration and collective action relate to the living planet and its interactions with the broader Earth system, like climate dynamics, soil formation or the water cycle.

Here, we are inspired by David Grinspoon's call to move from an immature to a mature Anthropocene**—**to awaken to our role as a force of planetary change, to grow into this task and become conscious shapers of our life-supporting environment and caretakers of Earth's biosphere, to become graceful planetary stewards by collaborating and performing conscious stewardship of our own future on Earth [59]. A mature Anthropocene may require a new imagined order with purpose, meaning and pathways towards a livable planet and sustainable future which, at its core, recognizes that the biosphere of planet Earth is the only place in the Universe where we know for certain that complex life exists. All humans and our diverse civilizations have emerged within the biosphere, are part of it and coevolve with it. Our economies, societies and cultures are embedded within the biosphere. The biosphere is our home [4,60]. The following elements provide the foundation for a network of shared stories for a new imagined order for the Anthropocene.

### (a) Globally intertwined

Human groups and cultures have throughout history significantly shaped and been shaped by the biosphere [61–63]. During the industrial era, and the Great Acceleration of the past seven decades, in particular, the aggregated human imprint on Earth has rapidly become truly planetary and a dominant force of Earth system dynamics [64]. The Anthropocene biosphere is now one highly interconnected and entangled system of people and nature, or an intertwined, complex and coevolving social-ecological system [37,65].

### (b) The Anthropocene biosphere and the earth system

There is a dynamic interplay between the biosphere and the broader Earth system [66], i.e. with the climate, the atmosphere, the hydrosphere (water in the ocean, lakes, ground and air), the lithosphere (Earth's crust and the uppermost mantle, including the tectonic plates) and the cryosphere (water in solid form like ice sheets and caps, snow and ice cover, glaciers and frozen ground, including permafrost). Humankind have become the major force in shaping this interplay. Great progress has been made in terms of health, living conditions, communication and material well-being for billions of people but at the expense of erosion of natural capital and biosphere resilience [67]. This is reflected in e.g. human-induced global warming, melting of ice sheets, alterations of rainfall patterns and circulation, of biogeochemical cycles, of extreme events, ocean acidification, reduced resilience to tipping points, soil erosion, simplifications of landscapes and seascapes, and loss of biodiversity [1,25,68].

### (c) A safe operating space

The major expansion of the human dimension into a globalized world and becoming a significant planetary force in the operation of Earth system seems now to have pushed humankind out of the favourable Holocene epoch of the past eleven thousand years. During the unusual climate-stable Holocene, it was possible for agriculture and civilizations to materialize, and humans became a real player in biosphere dynamics [69,70]. Now, humanity seems to have rapidly expanded out of an epoch that has served humanity well, into the new trajectory of the Anthropocene—‘the age of humankind'—moving humanity into unknown terrain. The Grinspoon [59] challenge of conscious stewardship now calls for navigating human actions and societies towards a safe operating space within planetary boundaries [71,72].

### (d) Active biosphere stewardship

For this to happen, it will not only be critical to curb human-induced climate change but also to enhance the regenerative capacity of the biosphere, and its diversity, to support and sustain societal development, to collaborate with the planet that is the home of humankind and to collaborate in a socially just, insightful, caring, trustful and sustainable manner [73–75]. Stewardship has a temporal dimension encompassing care for the future; an environmental dimension entailing care for the earth and other species; and an equity dimension focusing on a fairer distribution of resources, rights, responsibilities and power across society, within and among nations [2].

Stewardship, with incentives and norm shifts for managing and governing the biosphere, is a step in the transition towards a new imagined order, a meta-imagined order, almost like a cosmic order of deep appreciation of experiencing and sharing life in a unique place in an immense Universe. It is about stewarding our diverse human aspirations and actions in a fashion that plays in concert with the biosphere in which human societies are embedded and with which all people, irrespective of place, culture, or belief system, are deeply entangled. In this sense, diverse human societies are part of the planet's complex evolving system dynamics pattern. Societies can govern themselves in diverse ways in relation to and as part of these patterns but likely cannot make the economy, society or culture independent of these patterns. Such a fully enearthed imagined order would imply that humans are beings in nature, irrespective of the diverse ways in which human societies and civilizations are organized and operated.

### (e) Inclusive and sustainable futures

Transforming towards sustainable futures will require broadening cultural membership by promoting new stories that resonate, inspire and provide hope centred on a plurality of criteria of worth and social inclusion [38,39] and, importantly, with a collective appreciation and recognition of our interdependence with the biosphere [5] and economic and political action based on that recognition. It involves anticipating and imagining futures and behaving and acting on those in a manner that does not lead to loss of opportunities to live with changing circumstances, but enhances those opportunities, i.e. builds resilience for complexity and change [76].

The ultimate justice is for the current generations to secure a livable biosphere for future generations [77,78]. This will require social and cultural reorganization and transformation [79]. Such transformations toward a new meta-imagined order involve multiple elements, including agency, practices, behaviours, incentives, institutions, beliefs, values and world views and their leverage points at multiple levels [80,81]. Such shifts will require inclusive, collaborative approaches and trust-based institutions as well as collective action. They will require appreciation of practices, technologies, innovations, competencies and skills that help revitalize biosphere resilience [4].

## 5. Conclusion

There are signs that transformative systemic change is slowly getting underway. Bringing nature into decision-making plays a critical role in this situation of transformative systemic change. In this context, we have focused on the role of imagined orders—networks of shared imaginaries—capable of supporting well-being for an envisioned human population of at least nine billion, within the capacity of the biosphere to sustain it.

It is indeed truly remarkable how a species prone to group dynamics and tribal behaviour, with so many diverse stories, has been able to create a globally connected and actively interacting world, with amazing collaboration in many areas of human activity and relations. These imagined orders and actions based on them have provided numerous people with increased health, wealth and prosperity. However, the significance of a resilient biosphere in sustaining and enhancing such progress has not been sufficiently appreciated in current imagined orders. Now, we are confronted with a new reality, the new emerging playing field of the Anthropocene. Which are the imaginaries that will enable us to transform our actions and thrive in the Anthropocene?

Here, we have emphasized the meta-imagined order of being embedded in the biosphere because being embedded in the biosphere is more than an imaginary, it is foundational. Past imaginaries have, in general, taken biosphere resilience for granted. As a matter of fact, no matter what stories, all humans of diverse communities, societies and civilizations, irrespective of preferences, beliefs or religion, have always been an embedded part of the biosphere. We humans don't live on the planet but are beings in the biosphere.

A key challenge for humans of diverse cultures and in diverse settings is to understand our new role in the present situation as a dominant force in the operation of the biosphere and act on it—hence, the stewardship challenge. The new emerging stories need to relate to the biosphere's reality if transformational change towards a sustainable future is to be fulfilled. It will require more than just reducing the impacts of production systems on our living planet. It will require a revitalization of human–nature relationships, where caring for our home in the universe will serve as a core attractor, a meta-imagined order, something akin to a new cosmic order. Biosphere stewardship is about caring. Caring is about relations, with people and with life. It is about recognition, appreciation and respect for craftmanship in creating purpose and meaning for nurturing the livability of the biosphere.

## Data Availability

This article has no additional data.

## References

[1] Díaz S et al. 2019 Pervasive human-driven decline of life on earth points to the need for transformative change. Science **366**, eaax3100. (10.1126/science.aax3100) 31831642

[2] Chapin FS et al. 2022 Earth stewardship: shaping a sustainable future through interacting policy and norm shifts. Ambio **51**, 1907-1920. (10.1007/s13280-022-01721-3) 35380347 PMC8982314

[3] Baumol WJ, Oates WE. 1988 The theory of environmental policy. Cambridge, UK: Cambridge University Press.

[4] Folke C et al. 2021 Our future in the anthropocene biosphere. Ambio **50**, 834-869. (10.1007/s13280-021-01544-8) 33715097 PMC7955950

[5] Schill C et al. 2019 A more dynamic understanding of human behaviour for the Anthropocene. Nat. Sustain. **2**, 1075-1082. (10.1038/s41893-019-0419-7)

[6] Haidt J. 2001 The emotional dog and its rational tail: a social intuitionist approach to moral judgment. Psychol. Rev. **108**, 814. (10.1037/0033-295X.108.4.814) 11699120

[7] Haidt J. 2012 The righteous mind: Why good people are divided by politics and religion. New York, NY Pantheon Books.

[8] Fukuyama F. 1995 Trust: The social virtues and the creation of prosperity. New York, NY: Free Press.

[9] Weber M. 2009 The Protestant ethic and the spirit of capitalism. New York, NY: Norton Critical Editions. (Introduction by Richard Swedberg.)

[10] Fukuyama F. 2006 The end of history and the last man. New York, NY: Simon & Schuster.

[11] Lumsden CJ, Wilson EO. 1981 Genes,mind, and culture: the coevolutionary process. Cambridge, MA: Harvard University Press.

[12] Boyd R, Richerson PJ. 1988 Culture and the evolutionary process. Chicago, IL: University of Chicago Press.

[13] Boyd R, Richerson PJ, Henrich J. 2011 The cultural niche: why social learning is essential for human adaptation. Proc. Natl Acad. Sci. USA **108**(supplement_2), 10 918-10 925. (10.1073/pnas.1100290108) PMC3131818 21690340

[14] Henrich J. 2015 Culture and social behavior. Curr. Opin. Behav. Sci. **3**, 84-89. (10.1016/j.cobeha.2015.02.001)

[15] Henrich J, Muthukrishna M. 2021 The origins and psychology of human cooperation. Annu. Rev. Psychol. **72**, 207-240. (10.1146/annurev-psych-081920-042106) 33006924

[16] Waring TM, Wood ZT. 2021 Long-term gene–culture coevolution and the human evolutionary transition. Proc. R. Soc. B **288**, 20210538. (10.1098/rspb.2021.0538) PMC8170228 34074122

[17] Taylor C. 2004 Modern social imaginaries. Durham, NC: Duke University Press.

[18] Arrow KJ. 1972 Gifts and exchanges. Philos. Publ. Aff. **1**, 343-362.

[19] Souleles DS, Archer M, Thaning MS. 2023 Introduction to special issue: Value, values, and anthropology. Econ. Anthropol. **10**, 162-168. (10.1002/sea2.12285)

[20] Bouchard G (dd.). 2013 National myths: constructed pasts, contested presents. New York, NY: Routledge.

[21] Hobsbawm E, Ranger T (eds) 2012 The invention of tradition. Cambridge, UK: Cambridge University Press.

[22] Johnson S, Acemoglu D. 2023 Power and progress: Our thousand-year struggle over technology and prosperity. London, UK: Hachette.

[23] Gangl K, Torgler B, Kirchler E. 2016 Patriotism's impact on cooperation with the state: an experimental study on tax compliance. Polit. Psychol. **37**, 867-881. (10.1111/pops.12294) 27980350 PMC5125400

[24] Harari YN. 2014 Sapiens: A brief history of humankind. New York, NY: Random House.

[25] Steffen W et al. 2018 Trajectories of the earth system in the Anthropocene. Proc. Natl Acad. Sci. USA **115**, 8252-8259. (10.1073/pnas.1810141115) 30082409 PMC6099852

[26] Fehr E, Schmidt KM. 1999 A theory of fairness, competition, and cooperation. Q. J. Econ. **114**, 817-868. (10.1162/003355399556151)

[27] Kahneman D, Knetsch JL, Thaler RH. 1986 Fairness and the assumptions of economics. J. Business **59**, S285-S300. (10.1086/296367)

[28] Ostrom E. 1998 A behavioral approach to the rational choice theory of collective action. Am. Polit. Sci. Rev. **92**, 1-22. (10.2307/2585925)

[29] Gintis H, Henrich J, Bowles S, Boyd R, Fehr E. 2008 Strong reciprocity and the roots of human morality. Social Justice Res. **21**, 241-253. (10.1007/s11211-008-0067-y)

[30] Gintis H et al. 2005 Moral sentiments and material interests: the foundations of cooperation in economic life, vol. 6. New York, NY: MIT Press.

[31] Lieberman MD. 2013 Social: Why our brains are wired to connect. Oxford, UK: Oxford University Press.

[32] Ostrom E. 2000 Collective action and the evolution of social norms. J. Econ. Perspect. **14**, 137-158. (10.1257/jep.14.3.137)

[33] Wilson DS, Madhavan G, Gelfand MJ, Hayes SC, Atkins PW, Colwell RR. 2023 Multilevel cultural evolution: from new theory to practical applications. Proc. Natl Acad. Sci. USA **120**, e2218222120. (10.1073/pnas.2218222120) 37036975 PMC10120078

[34] Ambrose SH. 2010 Coevolution of composite-tool technology, constructive memory, and language: implications for the evolution of modern human behavior. Curr. Anthropol. **51**(S1), S135-S147. (10.1086/650296)

[35] Lee EA. 2020 The coevolution: the entwined futures of humans and machines. New York, NY: MIT Press.

[36] Berkes F, Folke C. 1998. Linking social and ecological systems for resilience and sustainability. In Linking social and ecological systems. Management practices and social mechanisms for building resilience (eds F Berkes, C Folke, J Colding), pp. 1-25. Cambridge, UK: Cambridge University Press.

[37] Reyers B, Folke C, Moore M-L, Biggs R, Galaz V. 2018 Social-ecological systems insights for navigating the dynamics of the anthropocene. Annu. Rev. Environ. Resour. **43**, 267-289. (10.1146/annurev-environ-110615-085349)

[38] Lamont M. 2019 From ‘having’ to ‘being’: self-worth and the current crisis of American society. Br. J. Sociol. **70**, 660-707. (10.1111/1468-4446.12667) 31190392

[39] Pascual U et al. 2023 Diverse values of nature for sustainability. Nature **620**, 813-823. (10.1038/s41586-023-06406-9) 37558877 PMC10447232

[40] Gadgil M, Berkes F, Folke C. 1993 Indigenous knowledge for biodiversity conservation. Ambio **22**, 151-156. 10.1007/s13280-020-01478-7 PMC8035379 33566330

[41] Garnett ST et al. 2018 A spatial overview of the global importance of Indigenous lands for conservation. Nat. Sustain. **1**, 369-374. (10.1038/s41893-018-0100-6)

[42] Tengö M, Hill R, Malmer P, Raymond CM, Spierenburg M, Danielsen F, Elmqvist T, Folke C. 2017 Weaving knowledge systems in IPBES, CBD and beyond: lessons learned for sustainability. Curr. Opin. Environ. Sustain. **26–27**, 17-25. (10.1016/j.cosust.2016.12.005)

[43] Milkoreit M. 2017 Imaginary politics: climate change and making the future. Elementa: Sci. Anthropocene **5**, 62. (10.1525/elementa.249)

[44] Bouchard G et al. 2017 Social myths and collective imaginaries. Toronto, Canada: University of Toronto Press.

[45] Graeber D, Wengrow D. 2021 The Dawn of everything: A New history of humanity. London, UK: Allen Lane.

[46] Piketty T. 2020 Capital and ideology. Cambridge, MA: Harvard University Press.

[47] Olson M. 1993 Dictatorship, democracy, and development. Am. Polit. Sci. Rev. **87**, 567-576. (10.2307/2938736)

[48] Barrett S et al. 2020 Social dimensions of fertility behavior and consumption patterns in the Anthropocene. Proc. Natl Acad. Sci. USA **117**, 6300-6307. (10.1073/pnas.1909857117) 32165543 PMC7104011

[49] Piketty T. 2014 Capital in the twenty-first century. Cambridge, MA: Harvard University Press.

[50] Meadows D, Randers J, Meadows D. 2004 Limits to growth: The 30-year update. White River Junction, VT: Chelsea Green Publishing.

[51] Tainter J. 1988 The collapse of complex societies. Cambridge, UK: Cambridge University Press.

[52] Centeno MA, Callahan PW, Larcey PA, Patterson TS (eds) 2023 How worlds collapse: what history, systems, and complexity can teach us about our modern world and fragile future. Milton Park, UK: Taylor & Francis.

[53] Harari YN. 2016 Homo deus: A brief history of tomorrow. New York, NY: Random House.

[54] Tegmark M. 2018 Life 3.0: being human in the age of artificial intelligence. New York, NY: Vintage.

[55] Anderies JM, Barfuss W, Donges JF, Fetzer I, Heitzig J, Rockström J. 2023 A modeling framework for World–Earth system resilience: exploring social inequality and Earth system tipping points. Environ. Res. Lett. **18**, 095001. (10.1088/1748-9326/ace91d)

[56] Lenton TM, Rockström J, Gaffney O, Rahmstorf S, Richardson K, Steffen W, Schellnhuber HJ. 2019 Climate tipping points—too risky to bet against. Nature **575**, 592-595. (10.1038/d41586-019-03595-0) 31776487

[57] Rockström J et al. 2023 Safe and just Earth system boundaries. Nature **619**, 102-111. (10.1038/s41586-023-06083-8) 37258676 PMC10322705

[58] Bennett EM et al. 2016 Bright spots: seeds of a good Anthropocene. Front. Ecol. Environ. **14**, 441-448. (10.1002/fee.1309)

[59] Grinspoon D. 2016 Earth in human hands: shaping our planet's future. London, UK: Hachette.

[60] Dasgupta P. 2021 The economics of biodiversity: the Dasgupta review. London, UK: HM Treasury.

[61] Redman C. 1999 Human impact on ancient environments. Tuscon, AZ: Arizona University Press.

[62] Ellis EC. 2015 Ecology in an anthropogenic biosphere. Ecol. Monogr. **85**, 287-331. (10.1890/14-2274.1)

[63] Ellis EC et al. 2021 People have shaped most of terrestrial nature for at least 12,000 years. Proc. Natl Acad. Sci. USA **118**, e2023483118. 33875599 10.1073/pnas.2023483118 PMC8092386

[64] Steffen W, Crutzen PJ, McNeill JR. 2007 The Anthropocene: are humans now overwhelming the great forces of nature. Ambio-J. Hum. Environ. Res. Manag. **36**, 614-621. (10.1579/0044-7447(2007)36[614:TAAHNO]2.0.CO;2) 18240674

[65] Clark WC, Harley AG. 2020 Sustainability science: toward a synthesis. Annu. Rev. Environ. Resour. **45**, 331-386. (10.1146/annurev-environ-012420-043621)

[66] Lenton T, Latour B. 2018 Gaia 2.0: Could humans add some level of self-awareness to Earth's self-regulation? Science **361**, 1066-1068. (10.1126/science.aau0427) 30213897

[67] Dasgupta P, Ramanathan V. 2014 Pursuit of the common good. Science **345**, 1457-1458. (10.1126/science.1259406) 25237092

[68] Nyström M, Jouffray J-B, Norström AV, Crona B, Søgaard Jørgensen P, Carpenter SR, Bodin Ö, Galaz V, Folke C. 2019 Anatomy and resilience of the global production ecosystem. Nature **575**, 98-108. (10.1038/s41586-019-1712-3) 31695208

[69] Turner BL, Clark WC, Kates RW, Richards JF, Mathews JT, Meyers WB (eds) 1990 The earth as transformed by human actions. New York, NY: Cambridge University Press.

[70] Vitousek PM, Mooney HA, Lubchenco J, Melillo JM. 1997 Human domination of Earth's ecosystems. Science **277**, 494-499. (10.1126/science.277.5325.494)

[71] Steffen W et al. 2015 Planetary boundaries: guiding human development on a changing planet. Science **347**, 1259855. (10.1126/science.1259855) 25592418

[72] Richardson K et al. 2023 Earth beyond six of nine planetary boundaries. Sci. Adv. **9**, eadh2458. (10.1126/sciadv.adh2458) 37703365 PMC10499318

[73] Folke C, Biggs R, Norström AV, Reyers B, Rockström J. 2016 Social-ecological resilience and biosphere-based sustainability science. Ecol. Soc. **21**(3), 41. (10.5751/ES-08748-210341)

[74] Enqvist JP, West S, Masterson VA, Haider LJ, Svedin U, Tengö M. 2018 Stewardship as a boundary object for sustainability research: linking care, knowledge and agency. Landscape Urban Plan. **179**, 17-37. (10.1016/j.landurbplan.2018.07.005)

[75] Brown K et al. 2019 Empathy, place and identity interactions for sustainability. Global Environ. Change **56**, 11-17. (10.1016/j.gloenvcha.2019.03.003)

[76] Berkes F, Colding J, Folke C. 2003 Navigating social-ecological systems: building resilience for complexity and change. Cambridge, UK: Cambridge University Press.

[77] Pearce D. 1987 Foundations of an ecological economics. Ecol. Modell. **38**, 9-18. (10.1016/0304-3800(87)90042-1)

[78] Gupta J et al. 2023 Earth system justice needed to identify and live within earth system boundaries. Nat. Sustain. **6**, 630-638. (10.1038/s41893-023-01064-1)

[79] Carpenter SR, Folke C, Scheffer M, Westley FR. 2019 Dancing on the volcano: social exploration in times of discontent. Ecol. Soc. **24**, 1. (10.5751/ES-10839-240123)31798644

[80] Abson DJ et al. 2016 Leverage points for sustainability transformation. Ambio **46**, 30-39. (10.1007/s13280-016-0800-y) 27344324 PMC5226895

[81] Moore M-L, Milkoreit M. 2020 Imagination and transformations to sustainable and just futures. Elementa: Sci. Anthropocene **8**, 1. (10.1525/elementa.397)

